# Church-Sponsored Promotornan di Salú/Community Health Worker-Led Health Fair Promoting Hypertension Awareness in Willemstad, Curaçao: A Pilot Study Assessing Participant Satisfaction and Experience

**DOI:** 10.3390/ijerph22091318

**Published:** 2025-08-25

**Authors:** Kenneth E. Christopher, Jenna R. Krall, Tiffany Arvizu, Alice Juliet, Sinead Mathilda-Fraaij, Elisette Rooi-Cannister, Lona D. Bryan

**Affiliations:** 1 Department of Public Health, School of Health Sciences, Liberty University, Lynchburg, VA 24515, USA; tfarvizu@liberty.edu; 2 Department of Global and Community Health, College of Public Health, George Mason University, Fairfax, VA 22030, USA; jkrall@gmu.edu; 3 Iglesia House of Worship (HOW) Curaçao, Erosweg, Willemstad 44P7+FFG, Curaçao; alicejjuliet@gmail.com (A.J.); sinishama@yahoo.com (S.M.-F.); ecannister001@gmail.com (E.R.-C.); 4 Helms School of Government, Liberty University, Lynchburg, VA 24515, USA; ldbryan@liberty.edu

**Keywords:** community health worker, health fair, participant satisfaction, participant experience, Promotornan di Salú, CHW-led health fair, health promotion

## Abstract

High blood pressure, or hypertension, remains a leading risk factor for cardiovascular disease, contributing significantly to global morbidity and mortality, particularly in Caribbean island nations like Curaçao. This pilot study assessed the impact of a health fair led by Community Health Workers (CHWs) or Promotornan di Salú and organized in collaboration with local faith-based organizations to increase hypertension awareness and promote preventive behaviors. The study utilized a cross-sectional design, and data were gathered from attendees at a health fair held on 29 June 2024, at the Iglesia House of Worship in Willemstad, Curaçao. A structured questionnaire was used to collect demographics, event satisfaction, health behavior intentions, and qualitative feedback data from participants aged 12 years and older. Of the 145 participants, 78.6% rated the event as excellent, 83.4% expressed plans to change their health behaviors, 80.6% intended to share information with family and friends, and 59.7% intended to follow up with a general practitioner (GP)/doctor. These findings highlight the effectiveness of culturally tailored, community-based initiatives to raise hypertension awareness, improve health literacy, and promote preventive health behaviors. The success of this intervention emphasizes the potential of CHW-led health fairs as valuable public health strategies and practical training opportunities for reducing the burden of chronic conditions like hypertension.

## 1. Introduction

High blood pressure, or hypertension, is a modifiable risk factor of cardiovascular disease (CVD) and is responsible for the global burden of morbidity and mortality worldwide, with an estimated ten million people dying annually from hypertension-related illness [1,2]. The Global Burden of Disease 2022 update indicated a 34.9% decline in age-standardized CVD mortality since 1990; yet, rates still span a sixfold variation, ranging from 73.6 to 432.3 deaths per 100,000 individuals, highlighting the enduring geographic disparities in health outcomes [3]. Hypertension is responsible for over 40% of all cardiovascular deaths worldwide, and the risk of major coronary or cerebrovascular incidents escalates dramatically, doubling with every 20 mm Hg rise in systolic pressure or 10 mm Hg increase in diastolic pressure beyond the critical threshold of 115/75 mm Hg [4]. The Global Burden of Disease 2022 analysis reaffirmed that high systolic blood pressure remains the single most significant modifiable contributor to cardiovascular disability and death, responsible for approximately 2565 Disability-Adjusted Life Years (DALYs) per 100,000 population [3]. About 1.39 billion people were diagnosed with hypertension in 2010, which equates to about 31% of adults globally [1]. Empirical data reveal a pronounced disparity in epidemiological trends between 2000 and 2010, characterized by a 2.6% decline in prevalence across high-income countries and a concurrent 7.7% rise in low- and middle-income countries (LMICs), illustrating the disproportionate global burden of disease [5]. Evidence shows that an estimated 1.28 billion adults aged 30–79 years old had a diagnosis of hypertension in 2019, and 82% lived in LMICs [2]. Awareness of blood pressure control and treatment is significantly low among these populations, and the burden of hypertension continues to rise worldwide [1].

High systolic blood pressure remains the single largest modifiable contributor to global CVD disability, accounting for approximately 2565 DALYs per 100,000 population [3]. Responding to this concerning trend, the World Health Organization set an ambitious target to achieve a 25% relative reduction in high blood pressure prevalence before the end of 2025, reinforcing the urgent need for improved awareness and intervention strategies [4]. Globally, in 2010, only 46.5% of hypertensive adults were aware of their condition, 36.9% were receiving antihypertensive medication, and just 13.8% had their blood pressure controlled, with awareness and control rates roughly half those figures in LMICs [5]. Researchers examining medical care for individuals with diabetes and hypertension in Mexico’s primary healthcare systems found that only 53% of treated hypertensive adults reached the established blood pressure targets [6]. They also identified previously undiagnosed hypertension in 15% of documented cases, revealing ongoing deficiencies in detection and management within LMIC health systems [6].

High blood pressure is a substantial risk factor for CVDs, including cerebrovascular disease and ischemic heart disease, and a modifiable risk factor for chronic kidney disease (CKD) and end-stage renal disease (ESRD) [7], which collectively are the leading causes of death among adults in Caribbean countries [8,9]. Factors that increase the risk for hypertension include genetic predisposition and family history, age, racial and ethnic background, and lifestyle choices, such as an unhealthy diet (high sodium and fat intake, low potassium), lack of physical activity, excessive alcohol consumption, smoking, poorly managed stress levels, and maintaining an unhealthy body weight [10,11]. There is emerging evidence showing that younger people, including young adults, adolescents, and children, are also at risk of hypertension because of unhealthy lifestyle factors [12,13]. Notably, in LMICs, the absolute burden of hypertension is most significant among adults aged 40–59 years, highlighting the loss of productive life-years [5].

Hypertension and CVD are significant disease burdens for morbidity and mortality in the Caribbean, including the island nation of Curaçao [14,15]. In Willemstad, Curaçao, hypertension is a growing concern, as about 1 in 5 adults have high blood pressure [15,16]. There are anecdotes suggesting there are numerous individuals in Curaçao who have high blood pressure and are unaware of their medical condition, and a significant number of people who have been diagnosed but do not have their blood pressure under control [15]. Individuals with uncontrolled hypertension are at higher risk for serious health complications, including stroke, CVD, and even death [17,18]. When properly managed and treated, the risks of hypertension can be minimized. Maintaining normal targeted blood pressure levels can reduce mortality from CVD and prevent CKD and ESRD conditions [7,16,19,20,21]. Population-based screening and detection of hypertension could enable early identification of individuals with high blood pressure, often before symptoms manifest, allowing for timely intervention and management [22,23]. Early detection and adequate treatment can significantly reduce the risk of severe complications associated with hypertension. Regular screenings help raise awareness about the importance of blood pressure management and encourage healthy lifestyle choices [22,23]. Costanzo et al. emphasized that implementing intensified prevention strategies tailored to local contexts, integrating lifestyle modification with cost-effective pharmacotherapy, is critical to mitigating the persistent global gap in blood pressure control, particularly in resource-constrained settings [4]. To minimize the widening disparity in blood pressure awareness, treatment, and control across regions, large-scale, cost-effective interventions, including salt reduction initiatives, health education campaigns, and community-based screening programs, must be urgently implemented in LMICs [5]. These approaches have yielded positive outcomes in high-income settings and offer scalable models for adaptation in LMICs, particularly across Caribbean nations such as Curaçao, to close the persistent gap in awareness, treatment, and control [5].

### 1.1. Faith-Based Organization and Health Promotion

Faith-based organizations, such as churches, occupy a central role in the social and cultural fabric of communities and have long played a vital role in promoting health and healthy behaviors within their communities [24,25,26]. These institutions are not only places of worship but also serve as community hubs that mobilize to provide health education, health screenings, support groups, community support services, and outreach programs that promote healthy lifestyles among their congregations and the broader community [24]. Often defined as a health ministry, these programs promote health and wellness by integrating spiritual care with practical health services to cultivate a comprehensive approach to well-being [25,26]. The programs enhance access to healthcare services by orchestrating health fairs, screenings, and vaccination initiatives [24,25,26]. Moreover, health ministry and health fair programs like those conducted in the USA establish clinical-community linkages to alleviate the burden of disease and improve the provision of preventive health services to vulnerable populations [24,25,26].

### 1.2. Promotornan di Salú (CHWs) in Curaçao as an Intervention

Chronic diseases like CVD and hypertension are among the new health challenges facing Curaçao in the twenty-first century, accounting for most deaths and increased healthcare costs [15,27]. Providing health education, promoting screening behaviors, and ensuring early disease detection are approaches to improve population health [22,28,29]. The Ministry of Health, Environment, and Nature Curaçao (GMN) has designated Community Health Workers (CHWs), or Promotornan di Salú, as an essential intervention in combating chronic illnesses, improving chronic disease care and health outcomes, and promoting health and wellness in Curaçao. In July 2024, the Ministry of Health (GMN) forged a collaborative partnership with faith-based organizations to establish an inaugural cohort of Promotornan di Salú to advance health promotion and wellness, early chronic disease detection, and management of long-term health conditions. By leveraging the social influence and network of churches, CHWs will use community health fairs to effectively promote hypertension awareness and prevention, early detection, healthy behaviors, and better control of blood pressure levels, thereby contributing to better health outcomes.

### 1.3. Health Fairs and Factors Influencing Participant Satisfaction

Health fairs are well-established, interactive community outreach initiatives designed to disseminate crucial health information, offer health screenings, and provide services in an accessible and engaging setting [28,29,30,31]. These events usually encompass free or low-cost screenings for high blood pressure, high cholesterol levels, diabetes, and vision and hearing problems [28,29,31,32]. They typically offer educational sessions on various health topics, including nutrition, physical activity, disease prevention, and interactive demonstrations on physical exercises and stress management techniques [29,30,31,32]. Health fairs provide valuable information regarding local health services, support groups, and other community resources and offer opportunities for healthcare professionals to practice their skills in a real-world setting [28,33,34]. These events are facilitated by a diverse array of healthcare providers, organizations, and vendors committed to promoting health and wellness within the community [32,35]. Meticulously planned and effectively executed health fairs are promising health promotion and wellness interventions that can engender positive health behavior change and enhance self-efficacy and chronic disease self-management skills [29,36,37,38,39,40]. There is evidence showing that health fairs are pivotal platforms to enhance access to preventive care, elevate health literacy, empower individuals with vital knowledge to adopt healthy behaviors [28,29,31,37,38,39,40,41,42,43], mitigate health disparities, and foster a culture of health and wellness within communities [28,29,31,37,39,40,42,43].

Participant satisfaction is a critical metric for evaluating the success and impact of health fair events [28,35,44,45,46]. This measure encompasses various aspects, including the quality and comprehensiveness of services, relevance of information, venue accessibility, positive interactions and engaging activities, and attendees’ overall experience [35,46,47,48]. High-quality, reliable health screenings, informative educational sessions, clear communication, and the provision of easily understandable health information enhance the credibility of the event and satisfaction levels [35,49,50,51,52]. Accessibility of the venue and placement in a familiar, community-centered, comfortable environment will likely encourage higher attendance [53,54,55,56]. A well-organized event with reduced wait times, clear signage, and effective logistics further enriches the experience and creates a positive experience [57,58,59,60]. High levels of participant satisfaction often correlate with increased trust and positive interactions between participants, health fair organizers, and health providers [28,35,46,61]. Integrating interactive and engaging activities can increase enjoyment and perceived value and improve overall participant satisfaction [62,63,64]. Surveys and feedback forms gathered during and after the event can yield significant insights into participants’ experiences, emphasizing successful aspects while pinpointing areas for enhancement [65,66,67]. The satisfaction of participants is a substantial metric for assessing the health fair’s impact on meeting the community’s health needs, fostering greater health awareness, and encouraging positive health behaviors [46,61,68,69].

### 1.4. Gaps in the Literature and Research Justification

A few studies have systematically examined the effectiveness and participant satisfaction of health fairs, revealing various insights into their impact on participant health outcomes [28,35,36,43,51,61,62,70,71,72]. These studies show that well-organized health fairs can promote health awareness, behavior changes, self-efficacy, and health literacy while identifying areas for improvement to enhance participant satisfaction and achieve better health outcomes [28,35,36,43,61,71,72]. Despite these findings, there is still a need for more research on the impact and participant satisfaction of health fairs, including health fair events sponsored by faith-based organizations. Although a few studies have examined faith-based health ministries [24,25,26], to the best of our knowledge, none have worked collaboratively with church leaders in an international setting to identify social and cultural issues related to hypertension, actively engaged church leadership in the design of the health fair intervention, and integrated church resources, like Promotornan di Salú (Community Health Workers) into the program. There is limited research on culturally sensitive health promotion programs tailored to diverse faith-based communities in international settings [73,74]. Curaçao’s unique cultural and religious landscape provides a valuable context for exploring how faith-based health fairs can address local health needs and improve participant satisfaction. Further research can provide insights into the benefits and challenges of health fairs in Curaçao, contributing to developing more effective and culturally relevant health promotion strategies. Understanding participant satisfaction in this context can help identify best practices for engaging diverse populations and fostering community support for integrating faith-based health initiatives into public health strategies to achieve better health outcomes [28,71,75].

### 1.5. Purpose and Objectives of the Study

The primary goal of this pilot study was to determine the perceived impact of the Promotornan di Salú (Community Health Workers)-led health fair on participants. The results of exit interview questionnaires were used to describe health fair participants’ satisfaction levels and the health fair’s effectiveness in influencing participants’ health knowledge, behaviors, intent, and habits. The health fair provided applied practice experience for the Promotornan di Salú training while promoting health and wellness among targeted church communities. We aimed to use the community health fair to increase awareness of the dangers of hypertension and cardiovascular diseases and promote health and wellness through health screenings and health education, including the provision of health information to educate individuals about the benefits of exercise and the consequences of inactivity and to motivate them to make positive health behavior changes. We hypothesized that participants would report a high level of satisfaction with the health fair and that the health education information provided by the Promotornan di Salú would improve participants’ health knowledge and inspire them to share information from the health fair with family and members of their communities and to routinely visit their General Practitioner (GP)/medical provider.

## 2. Materials and Methods

### 2.1. Design and Location

#### 2.1.1. Study Design

A cross-sectional research design was used to evaluate the perceived impact and effectiveness of a community health fair. A questionnaire was used to collect data on health fair participants’ opinions of activities at the health fair, including their level of satisfaction with health fair booths, health fair activities, and speaker preferences. Participants also used the questionnaire to self-report changes in their health knowledge and behaviors and their intent to seek follow-up care with a General Practitioner (GP) physician and share information from the health fair with family members, friends, or neighbors. We also assessed participants’ age, gender, marital status, reason for attendance, church affiliation, whether they registered to attend the health fair, and method of learning about the health fair to determine if these characteristics influenced their perceptions of the health fair.

#### 2.1.2. Location

This study was conducted on the Dutch Caribbean Island, Curaçao, an autonomous island nation within the Kingdom of the Netherlands [76]. Curaçao lies in the southern Caribbean Sea, about 55 km north of the Venezuelan coast [76]. It is a part of the Windward Islands in the Lesser Antilles and has a land area of 444 square kilometers [76] and an estimated population of 155,826 residents as of 2 September 2023 [77]. Curaçao is rich in cultural diversity with over 20 different nationalities and numerous ethnic and religious cultures, including Afro-Caribbean (the majority), Dutch, Hispanic, Latin American, Portuguese, South- and East-Asian, and Roman Catholics, Protestants, and Evangelicals comprising over 80% of religious practices among the population [76,77]. Curaçao is a multilingual country with Papiamentu (a unique blend of the languages of Dutch, Portuguese, Spanish, African languages, and English), Dutch, and English designated as the official languages, and Spanish is commonly spoken by the native population [76,77,78]. The government of Curaçao is a parliamentary democracy, with the executive power exercised by a Prime Minister and the seat of government located in the capital, Willemstad [76].

### 2.2. Setting, Health Fair Event, and Participants

#### 2.2.1. Setting

The community health fair was held from 10 A.M. to 2 P.M. on Saturday, 29 June 2024, at the Iglesia House of Worship Erosweg, Willemstad, Curaçao. The health fair was a collaborative community outreach project that brought together organizations and local resources to identify and promote awareness of chronic health conditions while building healthier, more knowledgeable, resilient communities through collegial partnerships and collaboration. The Ministry of Health, Environment, and Nature Curaçao, Krus Kòrá Kòrsou (Red Cross Curaçao), Iglesia House of Worship Curaçao, Iglesia Bida Nobo Curaçao (New Life Church), Arise Church Curaçao, and Liberty University partnered to conduct the health fair.

#### 2.2.2. Health Fair Event

The health fair targeted the congregations of the Iglesia House of Worship, Iglesia Bida Nobo, and Arise Church Curaçao, but was open to all interested members of the surrounding communities of Willemstad, Curaçao. The Iglesia House of Worship (HOW) Radio Station 90.9 FM served as a primary medium for disseminating promotional information about the health fair. Other marketing and promotional channels included announcements at churches, posting on the Iglesia House of Worship Facebook Page, WhatsApp notifications distributed to church membership networks, and printed flyers shared with church and community members. The health fair was inclusive and accessible to all community members, without requiring predefined attendance eligibility criteria. All individuals who presented at the health fair were welcomed without restriction and encouraged to participate freely in available activities.

The health fair program of services and activities included information on health topics and screenings for hypertension and blood glucose. Health fair booths provided displays and educational information on issues regarding (a) back health, (b) oral health care, (c) sleep disorder/apnea, (d) breast, cervical, and colon cancer, (e) diabetes, (f) cardiovascular health, (g) dengue vector-borne disease awareness, and (h) physical activity, fitness, and nutrition. Five (5) health screening stations were staffed by Promotornan di Salú trainees and Community Health Worker instructors who supervised their applied practice experience. At the health screening stations, the Promotornan di Salú trainees obtained height and weight measurements and calculated Body Mass Index (BMI), measured vital signs (body temperature, pulse/heart rate, respiratory rate, and blood pressure), and provided health educational presentations on hypertension and heart disease risk factors and engaging in regular physical activity to promote health and wellness. Two health seminars provided an opportunity to impact practical health, promote awareness, and build confidence among community members. A local general practitioner physician and a dentist organized and presented the seminars on infant and children’s health and oral health care practices. A mobile van from the LabdeMed medical laboratory provided blood sugar screenings and analysis. In addition, a special kids’ health and wellness segment was offered to engage children ages 4–12 years old in the health fair. Children had their body temperature, height, and weight measured. Interactive hands-on activities, games incorporating fitness challenges and health and wellness themes, dancing, outdoor physical activity demonstrations, face paintings, balloons, posters, free gifts, music, and healthy and nutritious fruits and snacks were provided to create a fair-like atmosphere for the kids.

#### 2.2.3. Participants

On the day of the health fair, all individuals expressing interest in health screenings were electronically registered by designated health fair volunteers. The registration protocol involved obtaining informed consent, collecting demographic data, and assigning a unique identification number to each participant to ensure confidentiality. Although the total number of health fair attendees was estimated based on crowd size, survey participants were specifically those who elected to receive health screenings during the event. Participation in the health fair was open to the general public; however, for this study, we analyzed quality assessment feedback exclusively from individuals who underwent health screenings, were at least 12 years of age, and spoke Papiamentu, Spanish, or English. Participants completed health screening activities, including having their height, weight, and body mass index calculated and their vital signs measured. Study participants also attended the hypertension and heart disease risk factors health educational presentations and consented to be interviewed as part of the evaluation of the health fair event. Due to the non-random nature of participant selection, findings from this study may not be generalizable to the broader population.

### 2.3. Data Collection Methods

#### 2.3.1. Health Fair Quality Assessment Questionnaire

To evaluate participants’ satisfaction and overall experience at the health fair, we interviewed participants using a health fair quality assessment questionnaire developed by Liu et al. [71] that we modified to align with the focus and aim of our research. The questionnaire was administered by Promotornan di Salú trainees, students, and alumni from Liberty University, Lynchburg, Virginia, who were all trained to administer the survey in paper format and manage four health fair quality assessment stations to conduct the interviews. At the health fair quality assessment stations, health fair attendees were invited to voluntarily participate in interviews after completing their health screenings, engaging in the fair’s programs and services, and checking out to leave the event. The interviews were administered in English, Spanish, and Papiamentu via volunteer translators from local churches. The structured nature of the interviews allowed for uniform data collection, and the in-person interaction facilitated clarification and follow-up questions when necessary.

To assess the quality and effectiveness of marketing the health fair and participants’ likelihood to attend another health fair in the future, interviewees were asked (a) to list reasons why they came to the health fair; (b) to indicate how they heard about the health fair; (c) to indicate if they registered to attend the health fair; and (d) to indicate if they would attend the health fair next year. Participants were asked to rate the health fair using options (excellent, fair, or poor) to measure their level of satisfaction and to select a “yes” or “no” response if they planned health behavior changes because of knowledge gained from their participation in the health fair. Interviewed participants were also asked to select options reflecting how they planned to use any information they received at the health fair. The response options included “do not plan to use the information”, “plan to read the pamphlets/brochures for my own benefit”, “plan to share information with family members, friends, or neighbors”, “plan to see a GP/doctor”, “found that I had a health problem I did not know about previously”, “found that someone in my family has a health problem we did not know about”, and “learned about one or more organizations and their services that I did not know about.” In addition, participants were asked to provide feedback about their favorite exhibitors, booths, health fair activities, and presentation speakers. The questionnaire items appear in Table 1.

#### 2.3.2. Data Analysis

The interview data were recorded on paper questionnaires, imputed to Qualtrics for transcription to an electronic format, and downloaded as a Microsoft Excel Open XML Format Spreadsheet file (XLSX) for import to Microsoft Excel 365 Analysis ToolPak (Microsoft Corporation, Redmond, Washington) for statistical analysis. Descriptive statistics (sample size and percentages) were computed to summarize demographic characteristics and participant responses. Free-response questions soliciting participants’ comments on their favorite components of the health fair were collected and analyzed qualitatively.

## 3. Results

### 3.1. Demographic Characteristics of the Survey Respondents

Of the estimated 225 health fair attendees, 145 participants completed the survey. Most participants were female (N = 115, 79.3%), attended HOW church (N = 97, 66.9%), and were either single (N = 65, 44.8%) or married (N = 43, 29.7%) (Table 2). Participants included individuals of all ages, ranging from 12 (minimum age for inclusion) to 90 years, roughly uniformly distributed (Table 2).

### 3.2. Participants’ Quality Assessment of the Health Fair

Participants generally heard about the fair through an announcement at their church (N = 97, 66.9%) and/or from a friend (N = 25, 17.2%) (Table 3). They came to the event because they were curious (N = 99, 68.3%), it was convenient (N = 46, 31.7%), it was free (N = 36, 24.8%), because they had family attending (N = 26, 17.9%), or because their church was coming (N = 22, 15.2%) (Table 4). Half of the participants had registered for the fair (N = 72, 49.7%), and almost all participants plan to attend next year (N = 141, 97.2%).

Most participants rated the fair as excellent (N = 114, 78.6%) and planned to change their health behaviors because of the fair (N = 121, 83.4%). Participants plan to share the information received from the health fair with their friends, relatives, or neighbors (N = 116, 80.6%), read the pamphlets/brochures provided (N = 106, 73.6%), and/or visit a GP or doctor (N = 86, 59.7%) (Table 5). Fewer participants indicated they learned about new health problems for themselves (N = 19, 13.2%) or their families (N = 9, 6.2%). Only one participant indicated they would not use information from the health fair.

### 3.3. Summary of Participants’ Feedback

Participants’ feedback about the health fair was overwhelmingly positive, with many offering praise and constructive suggestions. Many commended the event’s organization and smooth execution, with remarks like “Very organized” and “Great idea!” highlighting positive impressions. A significant number appreciated the well-structured nature of the event. Still, some participants noted areas for improvement, commenting that certain segments were “Too long” or specific stations were overly crowded. Despite these suggestions, the feedback included enthusiastic endorsements like “Went very well above excellent”, showcasing high satisfaction levels. A few participants even expressed interest in having more health fairs in the future, emphasizing the event’s value to the community. These insights suggest that although the health fair was generally well-received, there is room to enhance the experience by resolving concerns about the wait times between health fair stations and crowd management.

Feedback on specific components of the health fair was similarly enthusiastic. Participants frequently highlighted their favorite exhibitors and booths, particularly those focused on blood pressure checks, height and weight assessments, glucose or blood sugar monitoring, heart rate, and sleep apnea evaluations. Many even expressed overall satisfaction by stating they enjoyed “All” the booths, indicating a broad appreciation for the services offered. Additionally, comments about the seminar speakers and exhibitors that provided specific topic presentations were positive, with highlights including the infant children’s health seminar, dental care and oral health presentations, and a notable mention regarding sleep-related topics. Together, these detailed comments underscore the value participants placed on the health screenings, local vendors, and exhibitors’ participation, and the educational presentations at the event. Overall, the feedback underscored the health fair’s success in providing attendees with valuable health-related resources and services, leaving a lasting positive impression.

## 4. Discussion

### 4.1. Study Overview and Key Findings

This pilot study evaluated the perceived impact and effectiveness of a CHW/Promotornan di Salú-led health fair. The primary objectives were to assess participant satisfaction and determine whether the event improved health knowledge, motivated behavior change, and increased the likelihood of follow-up care. The survey results demonstrated that nearly 80% of participants rated the fair as excellent, over 83% reported intent to change their health behaviors, and almost all indicated they would attend future events. These findings underscore the potential of culturally tailored, community-based interventions to raise hypertension awareness and promote preventive health behaviors.

### 4.2. Interpretation and Comparison with Previous Research

Our findings, indicating high participant satisfaction (nearly 79% rating the event as excellent) and robust intentions for health behavior change (over 83% planning to modify their routines and more than 80% planning to disseminate health information), are consistent with prior studies examining the impact of community health fairs [28,29,31,35,37,38,39,40,42,43,61]. For instance, Allen et al. [61] demonstrated that free medical fairs can foster immediate behavioral intentions and improved health practices, echoing our observation of participants’ willingness to seek follow-up care and share health information. Similarly, Branford et al. [29] reported that community health fairs in low-resource settings enhance health literacy and promote preventive behaviors, paralleling our results regarding high overall satisfaction and the proactive use of health resources by attendees. The positive outcomes observed in this study are consistent with previous research indicating that well-organized health fairs can significantly improve health literacy, self-efficacy, and preventive behaviors [28,61]. However, our study extends these findings by incorporating a unique collaboration with faith-based organizations and integrating Community Health Workers (CHWs) in an international setting in the Dutch Caribbean, a strategy rarely documented in the literature. The CHW-led health fair served as a valuable applied practice experience, offering CHWs critical exposure to the public health and medical needs of the communities they will serve. This hands-on opportunity helped foster CHWs’ understanding and appreciation of their pivotal role in promoting chronic disease awareness and health and building trust within local populations.

Moreover, the health fair enabled CHWs to deliver targeted health education to individuals, many of whom presented with elevated blood pressure readings or risk factors for hypertension. Often unaware of their condition or associated risks, these individuals were encouraged to seek follow-up care with their GPs. Through these efforts, the health fair reinforced the importance of CHWs in addressing public health needs and demonstrated the effectiveness of health fairs in promoting overall community health. The event’s accessible venue, the trust fostered by church-based networks, and the cultural considerations for multilingual communication likely drove the high satisfaction and self-reported behavior change. In contrast to some studies that highlight logistical challenges as a barrier, our data suggest that a well-planned health fair, supported by strong community engagement, can overcome logistical challenges identified in some studies, even with minor issues like crowding.

Our findings also resonate with the work of Fritz et al. [39] and Escoffery et al. [28], who emphasized that well-organized health screening events facilitate access to care and positively influence participants’ health management. The significant proportion of our respondents planning to visit a GP or doctor (nearly 60%) reflects similar outcomes noted by Jobman et al. [46] and Lee et al. [37], where health fairs effectively catalyzed increased engagement with healthcare services. Moreover, studies by Lindgren et al. [43] and Murray et al. [31] stress the significance of event organization and logistical considerations in achieving high participant satisfaction, a finding corroborated by our study despite minor concerns regarding crowding and wait times.

Although 85% of health fair participant respondents were aged 31 years or older, only 29.7% reported being married. We are unable to definitively explain the observed discrepancy between participants’ age and marital status within the current dataset. We recognize that a complex interplay of cultural, socioeconomic, and contextual influences shapes marital status. Factors such as regional patterns in household composition or social affiliation, faith-based community dynamics, and individual life trajectories, including migration, caregiving roles, and evolving views on partnership, may all contribute to this demographic pattern. We encourage consideration of these broader dynamics in interpreting the findings and propose that future research explore such contextual influences more explicitly, particularly in studies aimed at understanding how sociodemographic variables interface with health-seeking behaviors in community and faith-based settings. Furthermore, it is noteworthy to mention that demographic distribution, particularly age, marital status, and church affiliation, may help inform CHW training and outreach strategies to ensure future health fair programming remains culturally responsive and aligned with the needs of participants.

Additionally, the culturally aware and faith-based framework of our intervention aligns with findings from Salman et al. [35], Yang et al. [38], and Kumpf et al. [40], which suggest that integrating community trust networks and tailored communication strategies can significantly enhance public health outcomes in diverse populations. Overall, the convergence of our results with the broader literature underlines the effectiveness of community health fairs as a strategic tool in promoting preventive care and awareness of chronic disease risk in underserved communities.

### 4.3. Strengths and Contributions

A strength of this study is its innovative approach: leveraging the social capital of faith-based organizations and integrating CHWs in a setting that reflects Curaçao’s diverse cultural landscape. This strategy enhanced the reach of the health fair and fostered a community-driven approach to addressing chronic diseases like hypertension. The study’s cross-sectional design, the application of a modified and validated questionnaire, and the inclusion and administration of a multilingual survey approach contribute to the findings’ reliability. This work advances public health research by addressing a gap in the literature regarding the impact and participant satisfaction of health fairs, specifically those sponsored by faith-based organizations in an international setting. This pilot study features faith-based organizations and CHWs’ critical role in hosting health fairs, emphasizing their capacity to foster trust, accessibility, and cultural awareness within communities. By investigating the health fair’s impact and participant satisfaction, our findings offer a holistic view of how these initiatives can enhance health literacy and effectively promote health education, prevention, and access to resources. Including participant satisfaction accentuates the importance of tailoring health interventions to meet community needs and expectations, ensuring that programs are impactful, well-received, and sustainable.

Our pilot study also underscores the value of CHW-led health fairs in promoting chronic disease awareness and management and enhancing preventive care within communities like Curaçao. By integrating CHWs into networks that support health and social care, these health fairs can promote chronic disease awareness, provide health education and services that resonate with diverse populations, and strengthen long-term strategies for managing the complex needs of individuals with chronic illnesses. Furthermore, our research brings to light the synergistic potential of combining health and faith-based initiatives, offering a nuanced understanding of how cultural and spiritual contexts could influence health outreach and community engagement. It provides a valuable framework to guide future public health activities and design strategies that harness the strengths of trusted community stakeholders, such as faith-based organizations and CHWs.

### 4.4. Limitations

Although the results are promising, several limitations warrant consideration. First, the cross-sectional design and use of self-reported data may have introduced recall or selection bias. Second, the small sample size does not adequately represent the overall population of Curaçao or the broader island nation communities within the Kingdom of the Netherlands, including Curaçao, Aruba, Sint Maarten (CAS), and Bonaire, Sint Eustatius, and Saba (BES). Third, as the data were informed by participants’ self-reports, potential inaccuracies may have arisen due to individual question interpretation, social desirability bias, or recall bias. Furthermore, the findings are constrained to a single health fair event. The sample was predominantly composed of middle-aged female participants from three churches, selected through convenience sampling. Although the health fair was open to the broader community, it likely did not attract a demographically diverse representation of the local population, making generalization of results difficult.

Another limitation of this study was the inability to track participants who, after exhibiting high blood pressure readings or risk factors for cardiovascular disease, were referred to their GP for follow-up care. Without follow-up data, it is challenging to determine whether participants acted upon these recommendations, thereby limiting our ability to assess another dimension of the health fair’s effectiveness in influencing health behaviors, intent, and long-term habits. This gap prevents robust assessment of the health fair’s downstream influence on participants’ health-seeking behavior, intent to change, and sustained lifestyle habits, thereby constraining conclusions about the health fair’s effectiveness beyond the immediate screening outcome. Future studies could integrate follow-up measures, such as post-event surveys and phone interviews, to assess referral adherence and subsequent health outcomes. Moreover, structured follow-up strategies, including automated text message reminders and CHW engagement, could facilitate objective verification of referral uptake and provide a clearer picture of the health fair’s long-term effectiveness in influencing health behaviors and impact on cardiovascular risk management.

Lastly, health fair attendees may have been particularly motivated and exhibited a greater-than-average interest in the event than the general population. The voluntary nature of participation may have favored respondents who were already positively inclined toward community events. Additionally, although the sample size was adequate for a pilot study, future research should involve more health fairs with extensive and diverse populations, including those in international settings, to improve generalizability. Alternative explanations for the high satisfaction rates, such as the general enthusiasm for community events rather than the intervention-specific effect, should be explored through further research.

### 4.5. Implications for Public Health Practice and Further Research

The findings from this study have important implications for public health practice, suggesting that integrating faith-based community networks with the expertise of CHWs can serve as an effective strategy in promoting community awareness of chronic disease conditions. Such community-centered initiatives are vital in culturally diverse and resource-constrained settings and could help mitigate limitations in traditional healthcare access. Moreover, this research suggests a viable model for promoting health and wellness and possibly reducing the burden of hypertension in underserved populations by demonstrating that community-based health fairs led by CHWs and supported by faith-based organizations can improve health knowledge and encourage preventive practices. Stakeholders, including public health officials and community leaders, should consider investing in and scaling up such culturally tailored interventions. Future research should aim to assess long-term health outcomes and refine event logistics to minimize issues like crowding and wait times.

Building on the descriptive findings of this pilot study, future research efforts could employ more rigorous methodological approaches to examine the influence of participant demographics and organizational involvement on health fair outcomes. For instance, incorporating inferential analyses, such as exploring associations between participant age, church affiliation, or registration status and self-reported intent to change health behaviors, may yield deeper insight into patterns of engagement and impact.

Moreover, controlled or comparative designs could be used to isolate the contributions of faith-based organizations to health promotion activities, thereby distinguishing their effects from those of secular or non-affiliated community efforts. Longitudinal follow-up of participants may also help assess the durability of behavioral change and health-seeking intentions following health fair events. Expanding the scope of future studies to include personal medical history, socioeconomic indicators, and health service utilization following health fair events would provide a more comprehensive understanding of needs and the efficacy of interventions. These enhancements could help support broader efforts to design equitable, culturally responsive, and evidence-informed community health strategies.

Although the present study provides valuable insight into the potential of faith-based collaborations to foster community trust and engagement, a design that incorporates secular comparison groups or evaluates multiple organizational models would allow for more precise attribution of observed effects. Future research may benefit from employing a controlled comparative design to more rigorously assess the influence of faith-based organizations on health fair outcomes. Such a design would bolster methodological rigor, strengthen the evidentiary basis for causal interpretations, and enhance the generalizability and scientific rigor of future findings. By isolating the unique contributions of faith-based frameworks to health promotion and service delivery, subsequent studies could more effectively inform best practices in the planning and implementation of community health interventions.

## 5. Conclusions

This pilot study demonstrates the effectiveness of CHW-led health fairs as a dual-purpose initiative, facilitating CHW education and community health promotion. CHW-led health fairs provide CHW trainees with hands-on experience and serve as a valuable platform for developing practical skills, reinforcing learned competencies, and fostering deeper community engagement. Beyond education, CHW-led health fairs are pivotal in promoting health, raising chronic disease awareness, and delivering preventive care services. The CHW/Promotornan di Salú-led health fair in Curaçao achieved high levels of participant satisfaction and demonstrated significant potential in improving health knowledge and promoting preventive behaviors related to hypertension. The overwhelmingly positive feedback and the strong intent to seek further care reiterate the health fair’s impact as a viable community and public health initiative.

Our findings also offer valuable insights for CHW practice, encouraging and empowering CHWs to actively participate in community-based interventions to bridge gaps in health literacy and foster informed decision-making. The success of this event underlines the importance of health fairs as a training opportunity, offering proof of concept for leveraging community engagement to enhance health outcomes and contribute to overall well-being. This study highlights the potential of collaborative, community-driven health promotion strategies to mitigate pressing public health challenges. The success of the health fair in fostering awareness, encouraging preventive behavior, and building community resilience paves the way for continued inquiry and innovation in public health interventions, ultimately contributing to the reduction of chronic diseases like hypertension-related morbidity and mortality in Curaçao and similar settings.

## Figures and Tables

**Table 1:** Health fair participants’ questionnaire content and response options.

Question Posed to Respondents	Response Options Provided
Gender (sex)	□ M □ F
Age	Write-in age (open-ended)
Your church	Write-in congregation name (open-ended)
Marital status	□ Single □ Partnered □ Married □ Separated □ Divorced □ Widowed
1. How would you rate the health fair in general?	□ Excellent □ Fair □ Poor Comments (open-ended)
2. Do you plan any changes in the things you normally do because of things you learned from or participated in at the health fair?	□ Yes □ No
3. How do you plan on using any of the health fair information received today? (Check all that apply)	□ I do not plan to use the information □ I plan to read the pamphlets/brochures for my own benefit □ I plan to share information with friends, relatives, or neighbors □ I plan to see a GP/doctor □ I found that I had a health problem I did not know about previously □ I found that someone in my family has a health problem we did not know about □ I learned about one or more organizations and their services that I did not know about
4. List your favorite exhibitors/booths/activities and speakers.	Write-in: My Favorite Exhibitors/Booths/Activities; My Favorite Speakers
5. Why did you come to the health fair? (Check all that apply)	□ Free □ Convenient □ Curious about health □ Recently felt bad □ My church came □ My family came □ I was in the area Other (write-in)
6. How did you hear about the health fair?	□ Radio (HOW 90.9) □ Live-stream church service □ Church announcement □ Poster/flyer □ Friend or neighbor □ Do not remember Other (write-in)
7. I registered to attend the health fair.	□ Yes □ No
8. I would attend a health fair next year.	□ Yes □ No

**Table 2:** Demographic characteristics (N, %) of the study sample (N = 145).

**Gender**	**N**	**%**
Male	30	20.7
Female	115	79.3
		
**Age**	**N**	**%**
12–20	13	9.0
21–30	7	4.8
31–40	20	13.8
41–50	19	13.1
51–60	33	22.8
61–70	26	17.9
71–80	20	13.8
81–90	7	4.8
		
**Marital Status**	**N**	**%**
Single	65	44.8
Married	43	29.7
Partnered	3	2.1
Widowed	21	14.5
Divorced	9	6.2
Separated	1	0.7
No Response Provided	3	2.1
		
**Church Affiliation**	**N**	**%**
House of Worship	97	66.9
Arise	7	4.8
Bida Nobo	7	4.8
Other	34	23.5

**Table 3:** Ways participants heard about the health fair (N, %).

	N	%
Church Announcement	97	66.9
Friend or Neighbor	25	17.2
Other *	22	15.2
Family Member	11	
Facebook	3	
Doctor Invite/Referral	1	
WhatsApp/Telephone App	2	
Work at House of Worship	3	
Colleague Referral	1	
Work with First Aid Station	1	
Radio (HOW 90.9)	21	14.5
Poster/Flyer	8	5.5
Live Stream Church Service	5	3.4

* Other was specified by free response and coded into these categories by two members of the study team. Participants were permitted to select more than one option.

**Table 4:** Reasons participants cited for attending the fair (N, %).

	N	%
Curious about health	99	68.3
Convenient	46	31.7
Free	36	24.8
My family came	26	17.9
My church came	22	15.2
Recently felt bad	13	9.0
Other	9	6.2
I was in the area	5	3.4

Note: Participants were permitted to select more than one option.

**Table 5:** How participants plan to use the health fair information (N, %) ^c^.

	N	%
I plan to share information with friends, relatives, or neighbors	116	80.6
I plan to read the pamphlets/brochures for my own benefit	106	73.6
I plan to see a GP/doctor.	86	59.7
I learned about one or more organizations and their services that I did not know about	39	27.1
I found that I had a health problem I did not know about previously	19	13.2
I found that someone in my family has a health problem we did not know about	9	6.2
I do not plan to use the information	1	0.7

^c^ One participant did not respond to this question (N = 144); participants were allowed to select more than one option.

## Data Availability

The original contributions presented in this study are included in the article. Further inquiries can be directed to the corresponding author.

## Abbreviations

The following abbreviations are used in this manuscript:

BES	Bonaire, Sint Eustatius, and Saba
BMI	Body Mass Index
CAS	Curaçao, Aruba, Sint Maarten
CHW	Community Health Worker
CKD	Chronic Kidney Disease
CVD	Cardiovascular Disease
ESRD	End-Stage Renal Disease
GMN	Ministry of Health, Environment & Nature, Curaçao
GP	General Practitioner
HOW	House of Worship
